# The impact of COVID-19 on implementation of mass testing, treatment and tracking of malaria in rural communities in Ghana: A qualitative study

**DOI:** 10.1371/journal.pone.0275976

**Published:** 2022-10-13

**Authors:** Ndong Ignatius Cheng, Philip Teg-Nefaah Tabong, Palmer Masumbe Netongo, Benedicta Ayiedu Mensah, Chuo Ennestine Chu, Effah-Baafi Yaw, Juliana Yartey Enos, Keziah Malm, Collins Stephen Ahorlu

**Affiliations:** 1 Faculty of Science, Department of Biochemistry, Catholic University of Cameroon, Bamenda, Cameroon; 2 Noguchi Memorial Institute for Medical Research, University of Ghana, Accra, Ghana; 3 Department of Social and Behavioural Sciences, School of Public Health, College of Health Sciences, University of Ghana, Legon, Ghana; 4 Navajo Technical University Crownpoint, Crownpoint, New Mexico, United States of America; 5 Molecular Diagnostics Research Group, Biotechnology Center, University of Yaounde I, Yaounde, Cameroon; 6 Ghana National Malaria Control Programme, Accra, Ghana; University of Leeds, UNITED KINGDOM

## Abstract

**Background:**

Mass test, treat and track (MTTT) of malaria is ongoing in the Pakro sub district of Ghana. In the delivery of MTTT of malaria, community health volunteers are trained to routinely provide this service through a door-to-door strategy. Following the report of the first cases of COVID-19 in Ghana, we conducted this study to explore the effects of the pandemic on the implementation of the MTTT of malaria intervention.

**Methods:**

Using qualitative methodology, we conducted ten focus groups discussions (FGDs) in eight communities: eight with community members (N = 49); one with health workers (N = 6), and one with MTTT of malaria volunteers. In addition, two in-depth interviews (IDI) were conducted, one with health worker and another with a health manager. All interviews were recorded, translated into English during transcription and analysed using QSR NVivo 12. Thematic content analysis was used in this study.

**Results:**

The findings of the study showed an increase in the number of people reporting with complications of malaria in health facilities in the study communities during the COVID-19 period. Some participants were of the view that COVID-19 rumours and misinformation could largely be responsible for the low coverage and uptake of the MTTT of malaria intervention. To sustain the uptake of the MTTT intervention, community engagement strategies were employed to identify and respond to these rumours. Also, incentive schemes were introduced to encourage parents and children to participate in the MTTT intervention during this period of COVID-19.

**Conclusion:**

Findings suggest that the COVID-19 pandemic has adversely affected the provision and uptake of malaria prevention and treatment services, especially the MTTT of malaria being implemented at the community level. These observations underscore the need to find innovative ways to address the challenges encountered in providing essential services during public health emergencies.

## Introduction

Malaria is a preventable condition of public health interest with high annual morbidity and mortality. Malaria is the most devastative infectious parasitic disease, killing more than one million people annually [1]. According to the World Health Organization (WHO) data for 2018, about 228 million cases of malaria and 405,000 deaths were reported worldwide, with Africa displaying the greatest number of cases and the highest mortality [2]. There were an estimated 14 million more malaria cases and 47,000 more deaths in 2020 compared to 2019 [1].

Malaria is endemic in Ghana and all the 30 million inhabitants are prone to malaria infection. Malaria accounts for 40% of all OPD attendance, with the most vulnerable groups being pregnant women and children under five [3]. Ghana malaria transmission is heterogeneous and differs along varying ecological zones. Parasite prevalence is ecologically seasonal, peaking in a single wet season (June–October) in the northern savannah area. Nevertheless, in both forest and coastal ecological zones, malaria parasite prevalence peaks twice in a year [4]. As a result, Ghana has rolled out a number of community-based interventions to help reduce the malaria burden such as distribution of long-lasting insecticidal nets (LLIN), test treat track (TTT) at health facilities and seasonal malaria chemoprevention (SMC) [3].

The 2018 Astana Global Conference on Primary Health Care emphasized the integration of community engagement into primary health care services. Community-based activities towards supporting the continuity of essential services, such as malaria prevention, diagnosis and treatment, with its distinctive capacities for health care delivery and social engagement has been prioritized across the world [5]. The health facility-based test, treat and track (TTT) was introduced by the World Health Organization as one of its strategies to reduce the burden of disease in endemic countries. However, it faces the problem of loss to follow-up of patients in Ghana [6,7]. Currently there is renewed interest in community-based mass test, treat and track (MTTT) [8]. MTTT involves parasitological testing of an entire population of a defined geographical area from door-to-door and treatment of confirmed positive cases with an appropriate antimalarial medicine at approximately the same time. This is an active case detection process aimed at improving coverage of treatment in a timely manner. MTTT targets asymptomatic individuals who may carry the parasite but are not ill [9]. In Ghana, this initiative is being implemented in some selected communities such as Abease Newsite, Fante Town, Zongo (Adjenase/Kweitey), Piem/Odumsisi, Adesa, Sacchi/Tabankro and Odumtokuro in the Eastern Region of Ghana. The implementation is being coordinated by the Noguchi Memorial Institute for Medical Research (NMIMR) of the University of Ghana [10]. This programme relies on the principles of community engagement to strengthen primary health care service in the continuity of malaria care. In this programme, trained volunteers provide MTTT services through a door-to-door testing of malaria using Rapid Diagnostic Test (RDT) and treatment using artemisinin-based combination therapy (ACTs). An earlier study on MTTT in Ghana underscored the effectiveness of the MTTT interventions in reducing the burden of malaria in rural settings [10]. If adopted, the MTTT programme could become one of the flagship malaria prevention and control interventions and contribute to malaria elimination. However, the success of these interventions depend on community members availability and acceptability which are often disrupted during public health emergencies [11] such as the COVID-19 pandemic, which was first identified in China in December 2019 [12], and declared a global pandemic by the World Health Organization (WHO) in March 2020 [13]. COVID-19 is caused by a novel human coronavirus, Severe Acute Respiratory Syndrome Coronavirus 2 (SARS-CoV-2). Ghana confirmed the first two cases of COVID-19 on 12^th^ March 2020. The incidence of cases has risen sharply across all regions of the country since then [14].

Malaria and COVID-19 have some common signs and symptoms which may include but not limited to: fever, breathing difficulties, tiredness and acute onset headache, which may lead to misdiagnosis of malaria for COVID-19 [15]. As has been reported COVID-19 has adversely affected the continuity of the Population Services International and the Global Malaria Community, such as seasonal malaria chemoprevention (SMC) and insecticide-treated bed nets (ITNs) distribution [15]. A decline in the number of participants in the MTTT interventions was observed at the onset of the COVID-19 pandemic. Disruptions to malaria control programmes have been linked to over 75 major resurgences in the past [16]. It is therefore important to document impact of the COVID-19 pandemic on ongoing malaria interventions such as MTTT of malaria. We therefore conducted this study to explore effects of COVID-19 on MTTT interventions in the Pakro sub-district of Ghana.

## Materials and methods

### Ethical approval

The protocol for this study was reviewed and approved by the Ethics Review Board of the Noguchi Memorial Institute for Medical Research (No. 068/19-20). All participants signed a written informed consent form before data elicitation.

### Study design

This study adopted narrative approach to qualitative research. Narrative research allows participants in a study to share their experiences in the community [17]. It was therefore adopted because the study was undertaken to document effects of the COVID-19 pandemic on the implementation of the ongoing MTTT intervention in the Pakro. Sub-district in the Eastern region of Ghana.

### Study area

The study was conducted in Pakro, one of the four sub-district of the Akwapim South district of the Eastern Region of Ghana [18]. The district has four Health Centres and 15 Community-based Health Planning and Services (CHPS) compounds that render community health care to residents. Malaria is the highest cause of morbidity and mortality among residents [19]. The Pakro Health Centre is one of thirty sentinel sites monitoring malaria prevalence in the country coordinated by the Noguchi Memorial Institute for Medical Research (NMIMR). Data available at the Pakro Health Centre show that malaria is the highest reported cases of the Outpatient department and is currently implementing MTTT interventions. Hence, this sub district was purposively selected for assessing the effect of COVID-19 pandemic on the implementation of MTTT.

### Selection of study participants

This study employed purposive sampling, a non-probabilistic sampling procedure [20]. Maximum variation purposive sampling was adopted to ensure that wide range of stakeholders participated in the study to reduce bias [21]. We identified participants who had experienced and participated in the MTTT intervention prior to and during the COVID-19 pandemic in Pakro sub-district and those who refused to participate in the MTTT intervention project, as required in purposive sampling technique [22]. We also selected health workers from the Pakro health Centre and community-based volunteers to participate in the study. The selection of varied groups of participants allowed us to performed triangulation during analysis [23].

### Data collection strategy

We used focus group discussions (FGDs) and in-depth interviews (IDIs) to collect the data in this study. Altogether, we conducted 12 FGDs; 10 among community members (N = 49), one among health workers (N = 6) and one among community-based volunteers (N = 6) who render the door-to-door MTTT services as part of the project. We complemented the FGDs with two in-depth interviews: one with a health manager (at Akwapim South Municipal Health District), and another with the health facility head of the community health facility). All FGDs and IDIs were conducted face-to-face whilst maintaining COVID-19 prevention protocols.

### Data collection tools

Semi-structured FGD and IDI guides were used to collect the qualitative data. The tools were designed in English language and pre-tested before data collection. The tools contained items such as knowledge about COVID-19; knowledge on protocols adopted to prevent COVID-19 during the pandemic; health seeking behaviour; and views about the MTTT project. The guide also contained items on effects of COVID-19 on the MTTT intervention project. The guides were translated during data collection. The translation was contextual rather than literal, meaning that questions were translated to convey the best meaning in local languages, these meanings were agreed on during pretesting.

### Data collection procedure

The FGD and IDI sessions took place at community level at a prearranged venue by community leaders and health workers in each community. FGD participants were made to sit in a semi-circle with the moderator and note taker sitting in front of the participants. The FGDs were conducted by trained Research Assistants working at the Noguchi Memorial Institute for Medical Research (NMIMR). These Research Assistants have prior experience in conducting qualitative interviews. The FGDs for community members were conducted in local language, Fante whilst that for health workers were conducted in English. During FGD, each participant was given the opportunity to respond to questions posed by the moderator before progressing to another question. This was done to ensure that all participated actively in the discussions. Participants were also allowed and encouraged to debate and negotiate their positions to reach consensus on topical issues. Inductive probing was done on emerging new areas. Each discussion was completed within 30–60 minutes. In-depth interviews were conducted in English language. Audio-recordings were replayed to study participants as a quality control measure in qualitative research [24]. The data was collected from July to August 2020.

### Data transcription and analysis

At the end of data collection, all voice recordings were translated from local languages to English during transcription. The transcripts were reviewed by comparing the translated versions with the original voice recordings. The researchers read through the transcripts noting the emerging issues. These were transformed into code book. The transcripts were imported into NVivo 12 software. The code-book was also imported into the software as nodes. Coding of each transcript was conducted within NVivo based on the code definition developed by the team in the codebook [25]. Thematic content analysis [20] was used to analyze the data. During coding, memos were also created for documenting our thoughts, doubts and insights that were emerging. Coded sections were regrouped into relevant categories and themes for presenting the results. The data was triangulated from different categories of participants to strengthen the findings [26] of the study, and representative direct quotations were used, to support the themes appropriately.

## Results

### Background characteristics of participants

Ten focus groups discussions (FGDs) were conducted in eight communities; eight with community members (N = 49), one among health workers (N = 6) and one among MTTT community volunteers (N = 6). The number of participants were limited in accordance with COVID-19 restrictions. In addition, in-depth interviews (IDI) were conducted with a health manager at the district health directorate and the health facility manager. Among the community members, males were 24 whilst females were 25 (Table 1).

**Table 1:** Distribution of study participants during focus group discussion and in-depth interviews.

Characteristic	number of participants	number of participants
	n (%)	n(%)
***Sex***		
Male	24(49)	8(57)
Female	25(51)	6(43)
**Total**	**49(100)**	**14(100)**
***Age group***		
≤20	4(8)	0
21–30	8(16)	5(36)
31–40	9(18)	8(57)
41–50	4(8)	0
>50	24(49)	1(7)
**Total**	**49(100)**	**14(100)**
***Level of Education***		
never been to school	9(18)	
Preschool/KG	5(10)	
primary	15(31)	
Junior secondary	11(22)	
Middle school	5(10)	
Senior Secondary	2(4)	
Vocational school/Tech after secondary	2(4)	
Vocational school/Tech after High School	0	6(43)
Graduate	0	5(36)
Postgraduate	0	3(21)
**Total**	**49(100)**	**14(100)**

### Knowledge on COVID-19

Participants in this study were generally aware of the COVID-19 disease in the country. Participants indicated the disease could be transmitted through close contact with infected individuals, contaminated hands, and respiratory droplets. Some participants shared their knowledge about COVID-19 as follows:

*“One can get COVID-19 through cough of infected person or shaking hands with infected person*. *They say we should practice social distance and not greet an infected person*. *Also*, *the infected person develops fever*, *catarrh and cough”*

(P6, FGD, Abease)

*“They also said we should not shake hands*, *we should also cover our nose and wash our hands”*

(P1, Adesa, Nsuablaso)

#### Recognition of signs and symptoms

On the recognition of signs and symptoms, participants mentioned cough, running nose, fever and general bodily weakness as follows:

*“We heard that COVID-19 is a new disease with some symptoms like malaria*, *catarrh*, *body pains*, *running nose”*

(P1, FGD Zongo)

Interviewees revealed that some of the signs and symptoms of the COVID-19 bear similarities with malaria. Hence the signs and symptoms of malaria could be misconstrued for COVID-19:

*Yes*, *because if you get malaria*, *you feel dizzy*, *weak and cold and it is the same with COVID-19”*

(P4, FGD, Piem Odumsisi)

Participants were also aware of the preventive protocols for COVID-19. Interviewees indicated washing of hands, the use of hand sanitizers and wearing of face mask as some of the practices that could protect people against COVID-19 as follows:

*They said an infected person can infect you through cough and touching infected surfaces*. *We need to sanitize our hands always*. *We must also avoid touching our face*, *nose and eyes*, *that is why we need to wear the face mask always”*

(P4, FGD, Volunteers)

*“On television*, *I heard that we should practice good personal hygiene and wash our hands regularly before touching or holding the things around us*. *They also said that we should use that medicine…eerr apetizer (Sanitizer) or what do you call it (laughing) to wash our hands”*

(P2, FGD, Piem Odumsisi)

### Fear of COVID-19 and stigmatization

The results showed that there was intense fear for COVID-19 due the similarities between the signs and symptoms of malaria and COVID-19. This was mentioned as one of the reasons for people refusing to seek for medical care when they have fever, headache, general bodily pains. To participants, health workers and community volunteers who deliver the door-to-door MTTT of malaria may refuse to attend to you because of similarities in the signs and symptoms between malaria and COVID-19. Some participants shared the views as follows:

*“When you have malaria*, *you cough but now if you cough*, *people associate it with COVID-19*. *So*, *we are afraid to go to come close to you even among community health volunteers and health workers in the hospital*. *The health workers would not attend to you because they will be thinking you have COVID-19”*

(P2, Adesa, Nsuablaso).

*“Going to the hospital nowadays has become something scary to us*. *Going to the hospital if I am sick will be very difficult*. *Just recently a pregnant woman was left unattended to at the hospital due to the fear of COVID-19 by the healthcare workers”*

(P3, Fante Town).

*“The nurses and the doctors nowadays don’t attend to us wholeheartedly like they used to do in the past*. *This is all because they fear that we might be having COVID”*

(P4, Fante Town).

However, some health workers indicated they were ready to provide care and treatment to community members despite fear of contracting COVID-19 as illustrated by one health care provider:

“*People in the community are afraid of COVID-19 but for us health workers we have trained to provide care*. *So*, *I am afraid of the condition but would continue to provide test and treat service to the community”*

### Rumours and misinformation on MTTT Programme

Community refusal to participate in the MTTT was also informed by the spread of COVID-19 rumours and misinformation. Interviewees confirmed widespread rumours that health workers were introducing COVID-19 virus through needle pricks during Rapid Diagnosis Test (RDT) for malaria, as part of MTTT interventions.

*“Many people were participating in the malaria testing*, *but at the onset of COVID-19*, *we heard rumours that some people will be going to villages to inject them with the virus*. *So*, *this might also account for the unwillingness of people to test for malaria”*

(P1, FGD, Fante Town)

Another rumour which led to declining community participation in the MTTT interventions was the belief that African countries were to be used to experiment COVID-19 vaccines. This rumour was widespread among community members. For example, two participants shared this misinformation as follows:

*“…because when COVID-19 came there was this rumour around that the vaccines have been developed and they are going to be tried on Africans [*…*] So going into this phase of MTTT*, *most of the community members were hesitant in taking part in this process*. *Some of them felt that it could have a link with the said Vaccines that they heard of”*

(IDI, HW 1)

*“These days the community members do not allow us to conduct the test because of the rumour that black race would be used to test the COVID-19 Vaccines*:

(IDI, HW2)

Despite the adoption and distributions of the Personal Protective Equipment (PPEs) to community members who agree to be tested for malaria, a rumour emerged that nose mask given to them by health workers have been infected with COVID-19 virus. A participant shared his experience on this as follows:

*“We heard rumours that we should not accept nose masks from people who come to distribute them to us*. *We heard that the nose masks could be infected with the virus and we will get sick*. *They said if we allow you to prick us*, *we will get the disease”*

(P3, FGD, Odumsisi)

### The impact of COVID-19 on malaria Mass Test Treat and Track (MTTT) interventions

Three main subthemes emerged as the effect of COVID-19 on the MTTT. These include: i) fear among health workers, which impacted their services, ii) delay in health-seeking among clients; and, iii) increase in severe malaria cases in health facilities.

#### Fear among health workers impacting their services

In view of the similarities in signs and symptoms of malaria and COVID-19, health workers become afraid when patients complain about experiencing headache, fever, general body pains, loss of smell and cough.

*“The nurses are sometimes afraid to come close to you*. *They are afraid the patient might have the disease and will infect them*. *It is difficult”*

(P4, FGD, Odumtokrom).

#### Delay in health-seeking among clients

Community members reported the fear of being diagnosed with COVID-19 as a reason for delays in care seeking, as rumors spread that these volunteers conducting the MTTT interventions were being used to conduct COVID-19 test as well. The use of infrared thermometers at health facilities in line with COVID-19 preventive measures accentuated this rumour, which reduced utilization of health services and negatively affected uptake of MTTT interventions,

*“Attendance at the health facility has reduced because people are scared to go to the facility for fear of contracting the disease*. *The person is sick but he or she will stay in the house*, *so probably it will become so severe before the person comes”*

(IDI, HW2).

*“You might be going to the hospital with your normal BP or fever*, *yet you can be diagnosed of COVID-19 and be sent away*. *So now we fear going to the hospital and to do the test for malaria as well”*

(P3, Adesa, Nsuablaso).

*“The previous thermometer we used was the armpit type*, *but now we use the thermometer gun*. *Immediately you bring out the thermometer gun*, *people think you have come to check for corona virus*. *They even say you send their results to the authorities and they will bring an ambulance to convey them away”*

(P1, FGD, Volunteers)

#### Increase in severe malaria cases in health facilities

The refusal of community members to take the malaria test as a result of the COVID-19 led to increased severe malaria cases reporting at health facilities in the community. Managers of the health care facilities in the study area revealed an increasing number of severe malaria cases among children in the hospital. This, they attributed to delays in health seeking and refusal to participate in MTTT. Some participants shared their views on this as follows:

*“There have been severe malaria cases especially in children since the onset of COVID-19 because most of the community people were told to stay at home and call If you had symptoms*. *So*, *in the case of malaria*, *some people had the severe case of malaria because they stay at home and did not seek treatment on time*. *They rushed to the health facility when it became worse”*

(P2, FGD, HW)

*“Although the person is sick*, *he or she will not come to the health facility because of how we will probably treat the person*. *So*, *they will stay in the house and come with severe malaria especially in the case of children”*

(IDI, HW 2)

### Impact of strategies adopted to increase uptake of MTTT during COVID-19 pandemic

Face masks and cakes of soap were distributed to study participants by the study team to mitigate the impact of COVID-19 on MTTT uptake. These provisions seem to have impacted positively on MTTT uptake. According to reports during the FGDs, these provisions encouraged community members to accept to be tested for malaria. One volunteer shared this in an FGD as follows:

*“After what we experienced with the people during the first testing (intervention)*, *we were given nose mask and soap to distribute to the people during the second testing (intervention)*. *And that made the work a bit simple*. *We had people cooperating”*

(P2, FGD, Volunteers)

In FGDs with community members, it emerged that the nose mask motivated people to agree to test for malaria. This made the participants feel that the project team cared as follows:

*“They came with nose masks and soap to share with those of us who agreed to test and gave medication to those who tested positive to malaria”*

(P1, FGD, Fante Town)

Though providing candies for children during the interventions was originally part of the project, the participants perceived this gesture in a positive light during the COVID-19 pandemic as follows:

*“The research team does well*. *Sometimes*, *they even come with candies to offer the little children who are tested*. *This makes the kids not to run away from them when they come”*

(P4, FGD, Fante Town)

### Discussions

This qualitative study was conducted to explore the effects of COVID-19 pandemic on the MTTT interventions in rural settings in Ghana. The ongoing COVID-19 pandemic places an extra burden on malaria control using the MTTT. A decline in MTTT uptake had been observed in participating communities of the ongoing MTTT intervention. The decline in uptake of the MTTT has resulted in an increase complicated malaria cases in health facilities in the community. As has been reported in many countries, particularly in sub-Saharan Africa which accounts for more than 90% of global malaria cases and deaths, many are facing a double challenge of protecting their citizens against existing threats to public health, such as malaria and emerging ones such as COVID-19. Disruptions to malaria control programmes have been linked to over 75 major resurgences in the past. Also, a study in Ghana showed that the COVID-19 caused a disruption to access to both facility and community-based public health interventions [27]. In addition, the COVID-19 pandemic has adversely affected access to emergency health care services in other countries [28]. Similar findings were reported during other public health emergencies like Ebola. In the 2014–2016 Ebola outbreak in West Africa it was reported that more people died from malaria than Ebola, including 7,000 additional malaria-associated deaths in children under five in Guinea, Liberia and Sierra Leone [16]. This underscores the need to find innovative ways to continue to provide essential health care services during public health emergencies.

From the study, it was clear that the decline in uptake of the MTTT was largely due to rumours regarding the clandestine use of the programme for COVID-19 testing as well as the deliberate spread of the infection by MTTT community volunteers and health workers. Rumours strive when people have no access to verified information. This calls for intense sensitization and education on COVID-19 in the community. These rumours have the potential to affect community trust for the health system which may undermine service delivery in the community. Across the world, there are reports of people refusing to participate in public health programmes such as polio vaccination campaigns due to rumors, such as the vaccine contains agents that could make people infertile or it was infected with human immunodeficiency virus [29,30]. Recently, rumors about health workers spreading Ebola virus led to violence and civil unrest in the Democratic Republic of Congo and Guinea with attack on health workers and mistrust towards outbreak response teams [31]. This underscores the need to incorporate rumour surveillance into public health interventions to identify and address these rumours at their early stages to reduce their impacts on programme implementations. Rumours about COVID-19 are widespread in Ghana [32].

In this study, community engagement to build stronger, sustainable relationships with the communities was adopted as a response strategy to demystify the misconceptions and rumours associated with the COVID-19 pandemic. This strategy was adopted because it has been reported as a very effective way to reduce the conceiving and spread of rumours during public health interventions [33].

### Conclusions

The study found that the COVID-19 pandemic adversely affected the uptake of MTTT interventions at the community level. This effect contributed to an increase in the number of people reporting with complicated malaria in health facilities. COVID-19 related rumours and misinformation were reported to be largely responsible for the low uptake of the MTTT interventions. These observations underscore the need to find innovative ways to address the challenges affecting access to health interventions during public health emergencies.
